# Hepatocyte-specific DDAH1 regulates fasting-induced hepatic lipid metabolism via modulating FABP1 expression and AMPK/mTOR-mediated autophagy

**DOI:** 10.1093/lifemeta/loaf042

**Published:** 2025-12-04

**Authors:** Kai Luo, Xiyue Shen, Siyu Wang, Fang Li, Yierxiati Jianggewaer, Weiping Sun, Zhongbing Lu

**Affiliations:** College of Life Science, University of Chinese Academy of Sciences, Beijing 101408, China; College of Life Science, University of Chinese Academy of Sciences, Beijing 101408, China; College of Life Science, University of Chinese Academy of Sciences, Beijing 101408, China; College of Life Science, University of Chinese Academy of Sciences, Beijing 101408, China; College of Life Science, University of Chinese Academy of Sciences, Beijing 101408, China; Department of Neurology, Peking University First Hospital, Beijing 100034, China; College of Life Science, University of Chinese Academy of Sciences, Beijing 101408, China

**Keywords:** DDAH1, hepatic steatosis, fasting, FABP1, AMPK

## Abstract

Under nutrient deprivation conditions, the liver maintains systemic energy homeostasis by mobilizing lipid reserves, a process often accompanied by hepatic lipid accumulation. Dimethylarginine dimethylaminohydrolase 1 (DDAH1), a key metabolizing enzyme for asymmetric dimethylarginine (ADMA), has been demonstrated to exert a protective effect in the pathogenesis of nonalcoholic fatty liver disease (NAFLD), yet its role in fasting-induced hepatic metabolic adaptation remains incompletely elucidated. In this article, we explored the function of DDAH1 in fasting-induced liver lipid accumulation using hepatocyte-specific *Ddah1* knockout (*Ddah1*^HKO^) mice. Compared with control mice (*Ddah1*^f/f^), *Ddah1*^HKO^ mice exhibited significantly attenuated hepatic steatosis after fasting. Lipidomic analysis of the liver revealed decreased levels of most lipid species (e.g., triglycerides and free fatty acids) in *Ddah1*^HKO^ mice. Further mechanistic studies demonstrated that *Ddah1* deletion downregulated the protein level of hepatic fatty acid binding protein 1 (FABP1) and activated the AMP-activated protein kinase (AMPK)/mammalian target of rapamycin (mTOR) signaling pathway, thereby enhancing autophagic flux and promoting lipid droplet degradation under fasting conditions. Hepatic overexpression of FABP1 reversed the anti-steatotic phenotype of *Ddah1*^HKO^ mice, while treatment with the AMPK inhibitor Compound C suppressed autophagy and increased hepatic lipid accumulation. In addition, overexpression of DDAH1 in hepatocytes exacerbated hepatic steatosis in fasted mice, coinciding with FABP1 upregulation and autophagy inhibition. Collectively, this article reveals that DDAH1 plays a critical role in hepatic lipid metabolism under fasting conditions by modulating FABP1 expression and AMPK/mTOR-mediated autophagy.

## Introduction

Under conditions of nutrient deprivation such as fasting or starvation, the liver, as the core organ of systemic energy metabolism, maintains whole-body energy homeostasis by mobilizing lipid reserves [1]. Metabolic adaptations to fasting lead to hepatic lipid accumulation, primarily resulting from enhanced lipolysis of adipose tissue, increased influx of free fatty acids (FFAs) into the liver, and a transient imbalance between hepatic lipid uptake and oxidative metabolism [2, 3]. Concurrently, the liver initiates ketogenesis, ­producing ketone bodies such as β-hydroxybutyrate (BDH) and ­acetoacetate, which serve as alternative energy sources for peripheral tissues (e.g., the heart and brain) during prolonged fasting [1, 4]. ­Moreover, fasting induces cellular autophagy—a highly conserved ­catabolic process that enables energy recycling. This includes the degradation of lipid droplets, a mechanism known as lipophagy, which plays a vital role in regulating liver lipid content and preventing excessive steatosis [5, 6].

Dimethylarginine dimethylaminohydrolase 1 (DDAH1) is a key metabolizing enzyme that degrades intracellular asymmetric dimethylarginine (ADMA) [7]. It is predominantly expressed in the liver and is a critical determinant of systemic ADMA levels [8]. Clinical studies have demonstrated that elevated circulating ADMA levels are closely linked to insulin resistance and impaired glucose metabolism in humans [9–11]; conversely, overexpression of DDAH1 decreases circulating ADMA levels and improves insulin sensitivity in mice [12]. While our previous work showed that hepatocyte-specific deletion of *Ddah1* aggravates high-fat diet (HFD)-induced hepatic steatosis via upregulation of S100A11 [13], the role of DDAH1 in fasting—a distinct metabolic state characte­rized by lipid mobilization rather than lipid overload—remained unaddressed, prompting the current investigation.

Fatty acid binding proteins (FABPs) constitute a key family of intracellular lipid-binding proteins. To date, no fewer than 10 FABP isoforms have been characterized, and each exhibits unique tissue distribution profiles as well as specialized functional properties [14]. Among these isoforms, FABP1 is abundantly expressed in the liver, where it makes up as much as 5% of the total cytosolic protein in hepatocytes [15]. By supporting the uptake, transport, and storage of fatty acids, FABP1 serves as a central regulator of hepatic lipid homeostasis and has also been linked to the development of metabolic disorders like nonalcoholic fatty liver disease (NAFLD) [15, 16].

Based on the abovesaid background, this article systematically investigated the role of DDAH1 in fasting-induced hepatic lipid accumulation by employing hepatocyte-specific *Ddah1* knockout (*Ddah1*^HKO^) mice. Our results showed that *Ddah1*^HKO^ mice exhibited significantly attenuated hepatic steatosis under fasting conditions, and this phenotype was associated with enhanced autophagic degradation of lipid droplets. In addition, this article confirmed that FABP1 may be a key downstream mediator of DDAH1 in regulating the hepatic metabolic response to fasting.

## Results

### Hepatocyte *Ddah1* is essential for fasting-induced hepatic lipid accumulation

**Figure 1:**
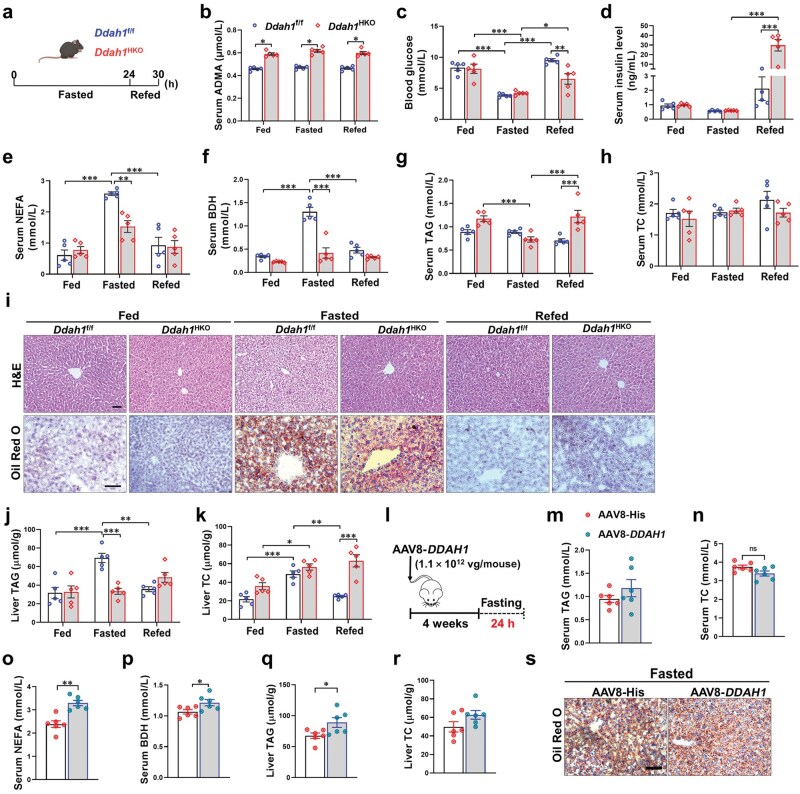
Hepatic DDAH1 promotes liver lipid accumulation in the fasted mice. (a) The time line of the experimental process. Ten-week-old male *Ddah1*^f/f^ and *Ddah1*^HKO^ mice were fasted for 24 h. In the refed group, mice were refed for 6 h after a 24-h fast. (b–h) Serum ADMA levels, blood glucose, and serum levels of insulin, NEFA, BDH, TAG, and TC in mice from three groups. (i) Histological changes and lipid accumulation in the livers assessed by H&E and Oil Red O staining, respectively. Scale bar = 50 μm. (j and k) Liver TAG and TC levels. (l) Flowchart of the experimental processes. Male C57BL/6 mice aged 8 weeks were intravenously injected with AAV8-His or AAV8-h*DDAH1* via the tail vein. After a 4-week interval, the animals were subjected to a 24-h fast and then sacrificed. (m–r) Serum TAG, TC, NEFA, and BDH levels and liver TAG and TC levels. (s) The effect of *DDAH1* overexpression on lipid accumulation in the fasted mice visualized by Oil Red O staining. Scale bar = 50 μm. Data are expressed as the mean ± SEM. ^*^P < 0.05; ^**^P < 0.01; ^***^P < 0.001.

*Ddah1* ^f/f^ and *Ddah1*^HKO^ mice were assigned randomly to fed, fasted, or refed groups, with the experimental timeline shown in Fig. 1a. Fasting for 24 h caused similar decrease in bodyweight in the genotypes (Supplementary Fig. S1a). Consistent with the role of DDAH1 as the key metabolizing enzyme of ADMA [7], serum ADMA levels were higher in *Ddah1*^HKO^ mice than *Ddah1*^f/f^ control mice across fed, fasted, and refed states (Fig. 1b); notably, fasting/refeeding did not alter ADMA levels in either genotype, indicating that DDAH1-mediated ADMA regulation is independent of nutrient status. Blood glucose levels decreased in both groups following fasting, and were notably elevated upon refeeding. No significant differences in blood glucose or serum insulin concentrations were observed between the two groups under either fed or fasted conditions. In contrast, after refeeding, *Ddah1*^HKO^ mice showed lower blood glucose levels alongside higher serum insulin levels (Fig. 1c and d).

Notably, the fasting-induced increases in serum non-esterified fatty acid (NEFA) and BDH levels were significantly greater in *Ddah1*^f/f^ mice than that in *Ddah1*^HKO^ mice, and this difference disappeared after refeeding (Fig. 1e and f). For serum triglyceride (TAG) levels, fasting and refeeding had no significant effect on *Ddah1*^f/f^ mice, but fasting decreased serum TAG levels in *Ddah1*^HKO^ mice, and refeeding increased them. After refeeding, serum TAG levels were significantly higher in *Ddah1*^HKO^ mice than that in *Ddah1*^f/f^ mice (Fig. 1g). Serum total cholesterol (TC) levels were not affected by fasting or refeeding in either group, with no significant differences observed between *Ddah1*^f/f^ and *Ddah1*^HKO^ mice (Fig. 1h).

As revealed by hematoxylin–eosin (H&E) and Oil Red O staining, fasting caused more obvious histological changes and lipid accumulation in the livers of *Ddah1*^f/f^ mice compared to *Ddah1*^HKO^ mice, and these differences vanished after refeeding (Fig. 1i). Consistently, after fasting, the hepatic TAG content in *Ddah1*^f/f^ mice was significantly higher than that in *Ddah1*^HKO^ mice. However, no notable difference in hepatic TAG levels was detected between the two groups following refeeding (Fig. 1j). Fasting induced a significant increase in hepatic TC levels in both groups, but refeeding only reduced hepatic TC levels in *Ddah1*^f/f^ mice, resulting in significantly higher hepatic TC levels in *Ddah1*^HKO^ mice than in *Ddah1*^f/f^ mice after refeeding (Fig. 1k).

To further verify the function of hepatocyte DDAH1 in fasting-induced hepatic steatosis, C57BL/6 mice were injected intravenously with Adeno-associated virus (AAV8)-TBG-h*DDAH1* through the tail vein (experimental workflow shown in Fig. 1l), with mice injected with AAV8-TBG-His serving as the controls. Results demonstrated that hepatocyte-specific overexpression of *DDAH1* significantly increased bodyweight as well as serum NEFA and BDH levels in fasted mice but did not affect serum TAG or TC levels (Fig. 1m−p; Supplementary Fig. S1b). In addition, *DDAH1* overexpression significantly increased hepatic TAG content in fasted mice (Fig. 1q) but had no significant impact on liver TC content (Fig. 1r). Oil Red O staining further confirmed that *DDAH1* overexpression exacerbated hepatic lipid accumulation in fasted mice (Fig. 1s). These findings indicate that DDAH1 in hepatocytes is a crucial regulator of hepatic lipid metabolism during fasting.

### DDAH1 significantly alters hepatic lipid species composition in fasted mice

**Figure 2:**
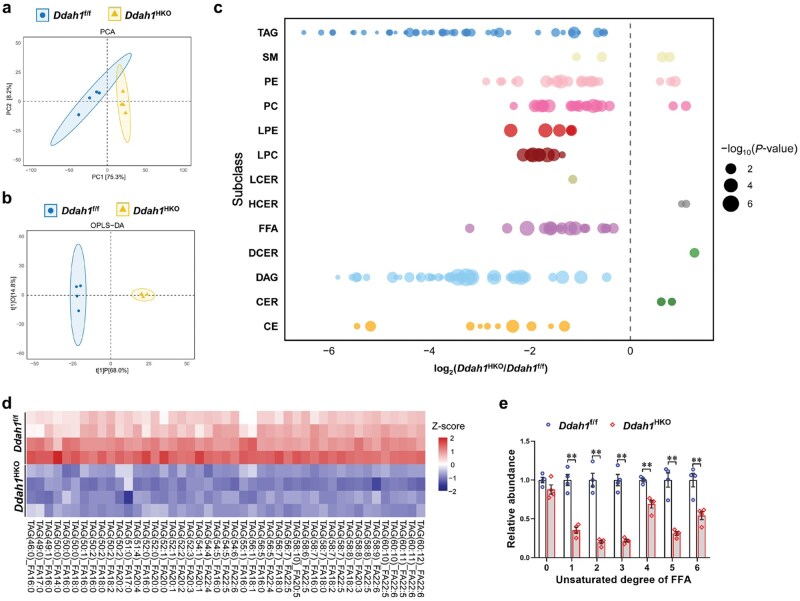
Hepatocyte-specific deletion of *Ddah1* affects the content of liver lipid species in the fasted mice. The livers from fasted *Ddah1*^f/f^ and *Ddah1*^HKO^ mice were subjected to lipidomic analysis. (a and b) Results from PCA and orthogonal projections to latent structures-discriminant analysis (OPLS-DA) presented as score scatter plots. (c) Fold changes of the primary liver lipid species in the fasted mice plotted in panels. All lipids depicted in this figure are statistically significant. (d) The relative levels of significantly altered TAG species presented as a Z-score heatmap. In this heatmap, data values are standardized to Z-scores, with the color of each cell indicating the Z-score of the corresponding data point. (e) The levels of unsaturated FFAs in the livers of fasted mice measured by lipidomics. Results are presented as mean values ± SEM. ^**^P < 0.01.

To further explore how DDAH1 influences hepatic lipid metabolism during fasting, lipidomic profiling was conducted on liver tissues from fasted mice. Score plots generated via principal component analysis (PCA) and supervised orthogonal partial least squares-discriminant analysis (OPLS-DA) revealed distinct differences in the major hepatic lipid species between fasted *Ddah1*^f/f^ and *Ddah1*^HKO^ mice (Fig. 2a and b). Specific changes in the main hepatic lipid species are shown in Fig. 2c. Compared with *Ddah1*^f/f^ mice, fasted *Ddah1*^HKO^ mice had significantly lower levels of most lipid species in the liver, including TAG, lysophosphatidylethanolamine (LPE), lysophosphatidylcholine (LPC), FFAs, diacylglycerol (DAG), and cholesterol ester (CE); in contrast, the levels of hexosylceramide (HCER), dihydroceramide (DCER), and ceramide (CER) were significantly increased. Heatmap analysis visually demonstrated the significant reduction in hepatic TAG subtype levels in fasted mice caused by hepatocyte *Ddah1* deficiency (Fig. 2d). ­Furthermore, hepatocyte *Ddah1* deficiency significantly decreased the content of unsaturated fatty acids in the livers of fasted mice—these fatty acids are important substrates for mitochondrial β-oxidation (Fig. 2e).

### Hepatocyte DDAH1 affects hepatic lipid metabolism-related pathways in fasted mice

**Figure 3:**
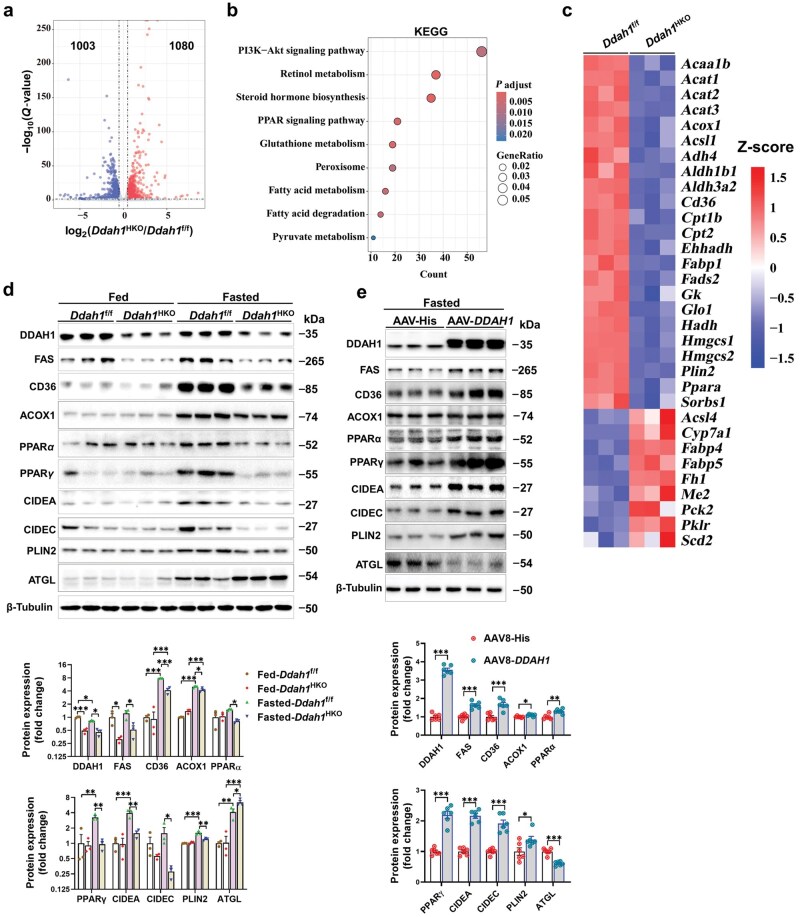
DDAH1 affects lipid metabolic pathways in the livers of fasted mice. (a) A volcano plot illustrating the fold changes of DEGs in the livers of *Ddah1*^f/f^ and *Ddah1*^HKO^ mice following a 24-h fast. (b) An advanced bubble chart presenting the KEGG pathways related to metabolism that were significantly enriched. (c) A Z-score heatmap depicting the expression profiles of genes involved in lipid metabolic pathways. (d) Western blot analysis performed on liver lysates from fed or fasted *Ddah1*^f/f^ and *Ddah1*^HKO^ mice. (e) Western blotting used to assess how *DDAH1* overexpression affects the protein levels of lipid metabolism-related factors in the livers of fasted mice. Values are shown as mean ± SEM. ^*^P < 0.05; ^**^P < 0.01; ^***^P < 0.001.

RNA sequencing (RNA-seq) was used to analyze and compare hepatic gene expression patterns between fasted *Ddah1*^f/f^ and *Ddah1*^HKO^ mice. This analysis identified 1003 genes with decreased expression and 1080 genes with increased expression, and a volcano plot was generated to visualize these differences in gene expression (Fig. 3a). Kyoto Encyclopedia of Genes and Genomes (KEGG) enrichment analysis revealed that these differentially expressed genes (DEGs) were mainly enriched in metabolism-related pathways, including retinol metabolism, peroxisome proliferator-activated receptor (PPAR) signaling pathway, fatty acid metabolism, fatty acid degradation, and pyruvate metabolism (Fig. 3b). A heatmap of genes involved in lipid metabolism-related pathways showed that the expression of several key fatty acid oxidation-related genes (*Acca1b*, *Acox1*, *Cd36*, *Cpt1b*, *Cpt2*, *Fabp1*, and *Ppara*) and ketogenesis-related genes (*Acat1/2/3* and *Hmgcs1/2*) was downregulated in the livers of fasted *Ddah1*^HKO^ mice (Fig. 3c).

As expected, DDAH1 protein levels were markedly decreased in the livers of *Ddah1*^HKO^ mice, whereas fasting exerted no notable impact on hepatic DDAH1 expression in either group. Fasting led to a marked increase in the hepatic protein levels of FAS, CD36, ACOX1, PPARγ, CIDEA, CIDEC, and PLIN2 in both groups; however, this elevation was significantly attenuated in *Ddah1*^HKO^ mice. In addition, hepatocyte *Ddah1* deficiency reduced PPARα protein levels and exacerbated the fasting-induced increase in ATGL protein levels in the livers of fasted mice (Fig. 3d). In contrast, injection of AAV8-TBG-h*DDAH1* into C57BL/6 mice increased hepatic DDAH1 protein expression by more than 3-fold in fasted mice. DDAH1 overexpression significantly increased the protein levels of FAS, CD36, ACOX1, PPARα, PPARγ, CIDEA, and CIDEC, while reducing ATGL protein abundance in the livers of fasted mice (Fig. 3e).

### DDAH1 regulates hepatic FABP1 protein expression and fatty acid uptake under fasting conditions

**Figure 4:**
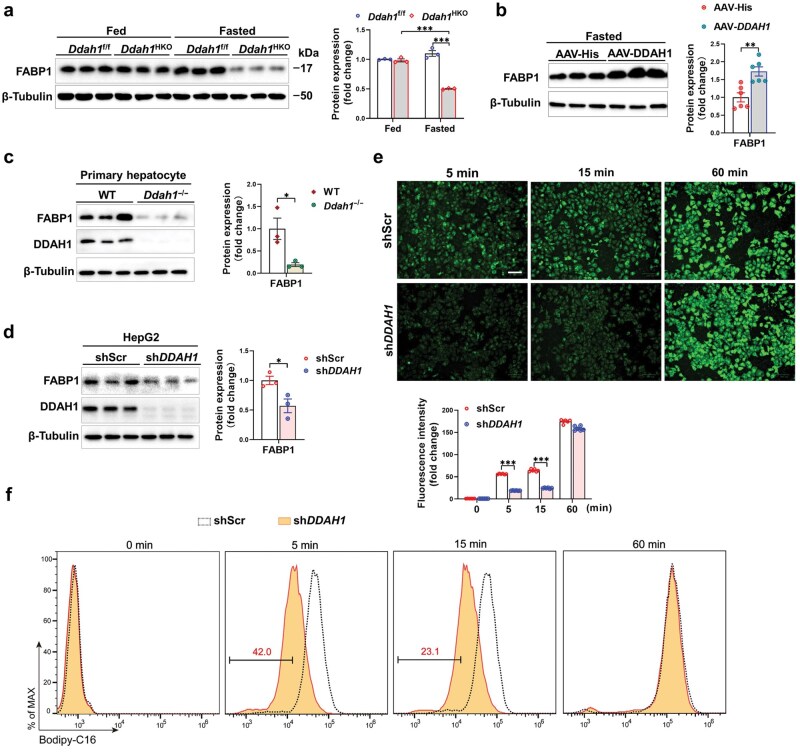
DDAH1 affects FABP1 protein level and fatty acid uptake in hepatocytes under fasting condition. (a) FABP1 protein levels in liver tissue from *Ddah1*^f/f^ and *Ddah1*^HKO^ mice under both fed and fasted states determined by western blot analysis. (b) The effect of *DDAH1* overexpression on hepatic FABP1 protein abundance in fasted mice evaluated using western blotting. (c and d) Western blot analysis of primary hepatocytes isolated from wild-type (WT) and *Ddah1*^−/−^ mice, and HepG2 cells stably transfected with shScr or sh*DDAH1*. The primary hepatocytes isolated from WT and *Ddah1*^−/−^ mice, along with HepG2 cells stably transfected with either a scrambled shRNA lentiviral vector (shScr) or a *DDAH1*-specific shRNA lentiviral vector (sh*DDAH1*), were cultured for 48 h in DMEM containing 1% glucose and 1% FBS. Cell lysates were then analyzed by western blotting. (e and f) Confocal microscopy and flow cytometry analyses of HepG2 cells stably transfected with shScr or sh*DDAH1.* HepG2 cells stably transfected with shScr or sh*DDAH1* were cultured in DMEM with 1% glucose and 1% FBS for 48 h, followed by incubation with BODIPY-C16 for varying durations. Confocal microscopy was used to capture images, and flow cytometry was employed to analyze fluorescence intensity. Scale bar = 100 μm. Data are expressed as mean ± SEM. ^*^P < 0.05; ^***^P < 0.001.

FABP1 plays a key role in promoting fatty acid uptake, intracellular transport, and delivery of mitochondrial β-oxidation substrates [15, 17]. Given that RNA-seq results showed downregulated *Fabp1* expression in the livers of fasted *Ddah1*^HKO^ mice, we further detected FABP1 protein levels. Results showed that hepatocyte-specific *Ddah1* deficiency exerted no notable impact on hepatic FABP1 protein abundance in mice under fed conditions. In contrast, under fasting conditions, FABP1 protein levels were markedly decreased in the livers of *Ddah1*^HKO^ mice (Fig. 4a). In addition, *DDAH1* overexpression significantly increased hepatic FABP1 ­protein levels in fasted mice (Fig. 4b).

To simulate fasting conditions, primary hepatocytes or HepG2 cells were cultured for 48 h in DMEM that contained 1% glucose and 1% fetal bovine serum (FBS). Results showed that either *Ddah1* deficiency in primary hepatocytes or *DDAH1* knockdown in HepG2 cells (mediated by shRNA) significantly reduced FABP1 ­protein levels under low-glucose and low-serum conditions (Fig. 4c and d).

To investigate whether the reduced FABP1 expression caused by *DDAH1* knockdown affects fatty acid uptake, control HepG2 cells (transfected with scramble shRNA, shScr) and *DDAH1*-knockdown HepG2 cells (transfected with *DDAH1*-specific shRNA, sh*DDAH1*) were cultured in DMEM containing 1% glucose and 1% FBS for 48 h, followed by incubation with fluorescently labeled fatty acid (BODIPY-C16) for different durations. Laser confocal microscopy observations revealed that compared with control cells, *DDAH1*-knockdown cells had significantly reduced fatty acid uptake at 5 and 15 min of incubation (Fig. 4e). Quantitative analysis by flow cytometry further confirmed that *DDAH1* knockdown significantly attenuated BODIPY-C16 uptake in HepG2 cells (Fig. 4f). However, following a 60-min incubation period, no statistically significant difference in fluorescence intensity was observed between the two cell groups.

### Hepatocyte-specific overexpression of FABP1 exacerbates hepatic lipid accumulation in fasted *Ddah1* ^HKO^ mice

**Figure 5:**
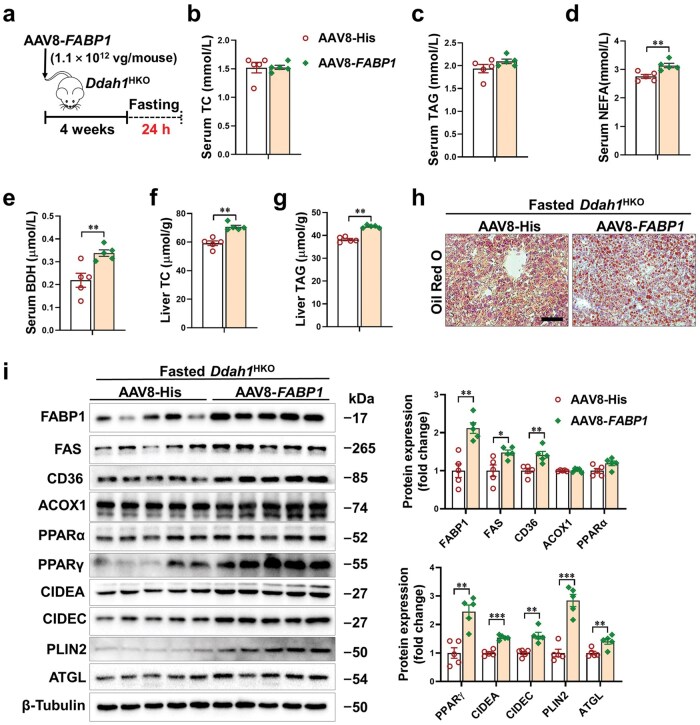
Overexpression of *FABP1* promotes hepatic steatosis in the fasted *Ddah1*^HKO^ mice. (a) Flowchart of the experimental processes. Ten-week-old male *Ddah1*^HKO^ mice received tail-vein injections of either AAV8-His or AAV8-*FABP1*. Four weeks later, the animals were fasted for 24 h and subsequently euthanized. (b–g) Serum levels of TC, TAG, NEFA, and BDH, as well as liver TC and TAG contents. (h) The effect of *FABP1* overexpression on hepatic lipid accumulation evaluated by Oil Red O staining. Scale bar = 50 μm. (i) Western blot analysis performed to assess the impact of *FABP1* overexpression on lipid metabolism-related proteins in the livers of fasted *Ddah1*^HKO^ mice. Data are presented as mean ± SEM. ^*^P < 0.05; ^**^P < 0.01; ^***^P < 0.001.

Given that fasting-induced hepatic steatosis is associated with downregulated hepatic FABP1 in *Ddah1*^HKO^ mice, this study injected AAV8-TBG-h*FABP1* into *Ddah1*^HKO^ mice via the tail vein (with AAV8-TBG-His injection as a control) to determine the effect of *FABP1* overexpression on the anti-steatotic phenotype of fasted *Ddah1*^HKO^ mice. The experimental workflow is shown in Fig. 5a. Results indicated that *FABP1* overexpression had no significant effect on bodyweight and serum TC or TAG levels in fasted *Ddah1*^HKO^ mice (Fig. 5b and c; Supplementary Fig. S1c), but significantly increased serum NEFA and BDH levels (Fig. 5d and e), as well as hepatic TC and TAG contents (Fig. 5f and g). As revealed by Oil Red O staining, *FABP1* overexpression significantly accelerated hepatic steatosis in fasted *Ddah1*^HKO^ mice (Fig. 5h). Western blot analysis revealed that injection of AAV8-TBG-h*FABP1* elevated hepatic FABP1 protein abundance by roughly 2-fold in fasted *Ddah1*^HKO^ mice, while concurrently significantly increasing the hepatic protein levels of FAS, CD36, PPARγ, CIDEA, CIDEC, PLIN2, and ATGL (Fig. 5i).

### DDAH1 regulates fasting-induced hepatic autophagy by inhibiting the AMPK/mTOR pathway

**Figure 6:**
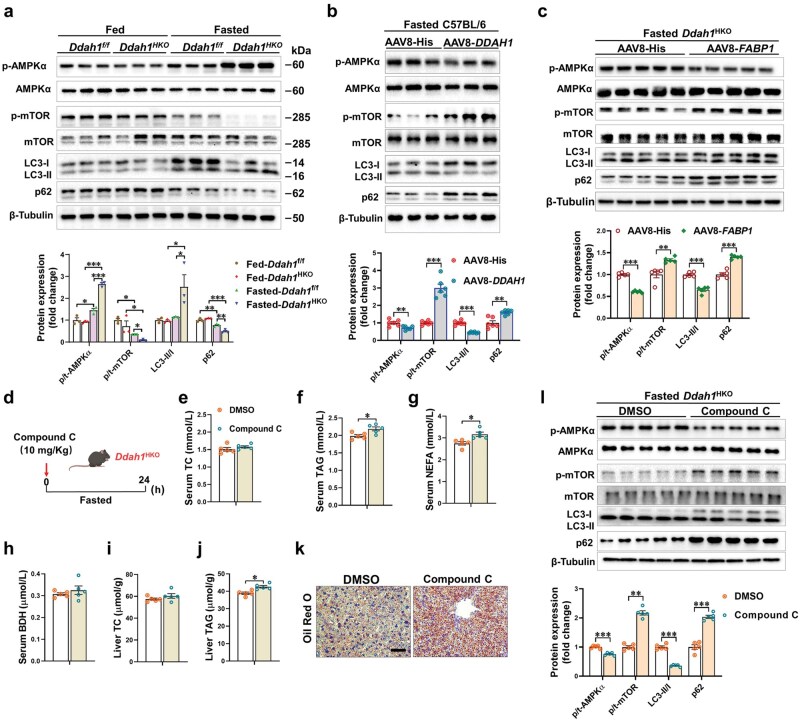
DDAH1 suppresses the AMPK/mTOR signaling pathway to block hepatic autophagy and facilitate liver lipid accumulation in fasted mice. (a and b) Western blotting analysis to examine the effect of hepatic *Ddah1* deficiency or overexpression on AMPK/mTOR/autophagy signaling. (c) *FABP1* overexpression affects AMPK/mTOR/autophagy signaling in the livers of fasted *Ddah1*^HKO^ mice. (d) Flowchart of the experimental processes. Ten-week-old male *Ddah1*^HKO^ mice were treated with 10 mg/kg Compound C via intraperitoneal injection and then fasted for 24 h. (e–j) Serum TC, TAG, NEFA, and BDH levels and liver TC and TAG content in each group. (k) Oil Red O staining to assess the impact of Compound C on liver lipid accumulation in the fasted *Ddah1*^HKO^ mice. Scale bar = 50 μm. (l) Western blot analysis used to examine the effect of Compound C on AMPK signaling pathways in the livers of fasted *Ddah1*^HKO^ mice. The values are presented as means ± SEM. ^*^P < 0.05; **P < 0.01; ***P < 0.001.

The induction of autophagy during fasting is tightly controlled by AMPK and mTOR signaling [18]. Western blot results showed that fasting significantly increased the phosphorylation of AMPKα (p-AMPKα) and decreased the phosphorylation of mTOR (p-mTOR) in the livers of both groups. Although hepatocyte *Ddah1* deficiency had no significant basal effect on the AMPKα/mTOR pathway, after fasting, *Ddah1*^HKO^ mice exhibited significantly higher hepatic p-AMPKα levels and lower hepatic p-mTOR levels than *Ddah1*^f/f^ mice. Consistently, hepatocyte *Ddah1* deficiency also significantly increased the LC3-II/LC3-I ratio and further reduced the level of the autophagic substrate p62 in the livers of fasted mice (Fig. 6a).

On the other hand, overexpression of *DDAH1* in fasted mice significantly reduced hepatic p-AMPKα levels and the LC3-II/LC3-I ratio, while increasing p-mTOR and p62 levels (Fig. 6b). In addition, overexpression of *FABP1* also significantly reduced p-AMPKα levels and the LC3-II/LC3-I ratio, and increased p-mTOR and p62 levels in the livers of fasted *Ddah1*^HKO^ mice (Fig. 6c).

To verify whether enhanced AMPKα phosphorylation contributes to the improved hepatic steatosis in fasted *Ddah1*^HKO^ mice, *Ddah1*^HKO^ mice were treated with 10 mg/kg of the specific AMPK inhibitor Compound C via intraperitoneal injection, followed by a 24-h fast (experimental workflow shown in Fig. 6d). Results showed that Compound C treatment had no significant effect on bodyweight and serum TC or BDH levels in fasted *Ddah1*^HKO^ mice, but significantly increased serum TAG and NEFA levels (Fig. 6e−h; Supplementary Fig. S1d); although hepatic TC levels were unaffected, hepatic TAG levels were significantly increased (Fig. 6i and j). The pro-steatotic effect of Compound C in the fasted *Ddah1*^HKO^ mice was also validated by Oil Red O staining (Fig. 6k). Western blot results showed that Compound C treatment significantly reduced p-AMPKα levels and the LC3-II/LC3-I ratio, while increasing p-mTOR and p62 levels in the livers of fasted *Ddah1*^HKO^ mice (Fig. 6l). However, Compound C administration did not alter the levels of FAS, PPARα, CIDEA, CIDEC, or FABP1 proteins (Supplementary Fig. S2).

Collectively, the results imply that DDAH1 acts as a negative regulator of hepatic autophagy under fasting conditions, and its deficiency promotes lipid clearance by enhancing autophagic flux.

## Discussion

This article uncovers a novel role of hepatocyte-specific DDAH1 in regulating hepatic lipid metabolism under fasting conditions and confirms that the DDAH1–FABP1–AMPK/mTOR–autophagy axis is a key regulatory pathway for maintaining hepatic lipid homeostasis. The data demonstrate that hepatocyte-specific *Ddah1* deficiency significantly attenuates fasting-induced hepatic steatosis, and this protective effect is primarily mediated through two complementary mechanisms: first, inhibiting fatty acid uptake and utilization by downregulating FABP1 expression; second, enhancing autophagic degradation of lipid droplets by activating the AMPK/mTOR signaling pathway.

Fasting primarily increases serum NEFA levels by stimulating lipolysis in white adipose tissue (WAT) [19]. Here, the results showed that fasting-induced serum NEFA elevation was significantly blunted in *Ddah1*^HKO^ mice, whereas hepatocyte-specific *DDAH1* overexpression further increased serum NEFA concentrations in fasted mice. Mechanistically, we confirmed that *Ddah1* deficiency reduced hepatic FABP1 protein abundance both in fasted mice and in hepatocytes cultured under low-glucose/low-serum conditions (a cellular model mimicking fasting). Critically, hepatic *FABP1* overexpression reversed the reduced serum NEFA phenotype in fasted *Ddah1*^HKO^ mice, which directly supports the notion that DDAH1 regulates hepatic NEFA uptake via FABP1. Given the well-established role of FABP1 as an intracellular lipid chaperone that facilitates fatty acid transport into hepatocytes [15], the downregulation of FABP1 in *Ddah1*^HKO^ mice likely impairs hepatic NEFA uptake, thereby decreasing the systemic demand for WAT-derived NEFA. In addition, we cannot exclude the possibility that hepatocyte-specific *Ddah1* deletion indirectly suppresses WAT lipolysis via altered secretion of hepatokines (e.g., fibroblast growth factor 21 and angiopoietin-like protein 4), which are known to regulate adipose lipid mobilization [20]. This hypothesis will be addressed in our subsequent studies to fully delineate the systemic metabolic effects of DDAH1.

FABP1 not only mediates fatty acid uptake but also acts as a ligand chaperone for nuclear receptors, including PPARα and PPARγ [14, 15, 17]. It delivers fatty acids and their derivatives (e.g., fatty acyl-CoA and eicosanoids) to these receptors, thereby activating the transcription of PPARα/γ target genes involved in fatty acid oxidation (e.g., *Acox1* and *Cpt1b*), ketogenesis (e.g., *Hmgcs2*), and lipid storage (e.g., *Plin2*) [15–17]. Consistent with this function, our RNA-seq and western blot data showed reduced levels of PPARα/γ target genes (e.g., *Acox1*, *Cpt1b*, and *Hmgcs1/2*) and proteins (e.g., ACOX1, PPARγ, and PLIN2) in fasted *Ddah1*^HKO^ mice—likely a direct consequence of FABP1 downregulation. Conversely, hepatic *DDAH1* overexpression upregulated these PPARα/γ targets, while *FABP1* overexpression in *Ddah1*^HKO^ mice reversed these changes and exa­cerbated hepatic steatosis. Collectively, these results confirm that the FABP1–PPARα/γ axis serves as a key downstream mediator through which DDAH1 regulates hepatic lipid metabolism under fasting conditions.

During fasting, a subset of incoming FFAs undergoes oxidation through the mitochondrial β-oxidation system. This process generates acetyl coenzyme A (acetyl-CoA), which in turn drives the formation of ketone bodies. The remaining FFAs can undergo reesterification to form TAG, which is subsequently deposited in the cytoplasm of hepatocytes [21]. Impaired hepatic β-oxidation—resulting from genetic deletion of *Pparα* [22] or *Cpt2* [23]—has been shown to exacerbate fasting-induced hepatic steatosis. Intriguingly, this article observed a paradoxical phenotype: although hepatic *Ddah1* deficiency in fasted mice attenuated fasting-induced increases in serum BDH levels, downregulated key ketogenic and fatty acid β-oxidation-related genes, and reduced hepatic unsaturated fatty acid levels (critical substrates for β-oxidation), these mice exhibited less hepatic lipid accumulation. This apparent contradiction can be explained by limited substrate delivery to hepatocytes: FABP1 downregulation not only reduces NEFA uptake but also impairs intracellular fatty acid trafficking to the mitochondria for oxidation. This interpretation is supported by our observation that restoring FABP1 in *Ddah1*^HKO^ mice increased ketogenesis markers (e.g., serum BDH) but also exacerbated steatosis—highlighting the pivotal role of FABP1 in balancing lipid uptake and oxidation under fasting.

Beyond regulating fatty acid uptake and utilization, DDAH1 also modulates hepatic lipid clearance via autophagy, a key adaptive mechanism for preventing excessive steatosis during fasting [24]. Autophagy (specifically lipophagy, the selective degradation of lipid droplets) releases FFAs from lipid droplets for β-oxidation, thereby limiting hepatic TAG accumulation [18, 25]. For example, genetic deletion of the Pellino 3 E3 ligase impairs autophagy-mediated lipid droplet clearance, exacerbating fasting-induced hepatic steatosis [26]. In our study, hepatic *Ddah1* deficiency in fasted mice increased the phosphorylation of AMPKα and the LC3-II/LC3-I ratio (a marker of autophagic flux), while decreasing the phosphorylation of mTOR and the level of p62 (an autophagic ­substrate). Conversely, hepatic *DDAH1* overexpression exerted the opposite effects on AMPK/mTOR signaling and these autophagy-related indicators. To confirm causality, we inhibited AMPK activity in fasted *Ddah1*^HKO^ mice using Compound C: this intervention suppressed autophagy (reduced LC3-II/LC3-I ratio and increased p62 level) and significantly increased hepatic lipid accumulation. These results collectively demonstrate that hepatic *Ddah1* deficiency mitigates fasting-induced hepatic steatosis in part by enhancing AMPK/mTOR pathway-mediated autophagy.

Notably, overexpression of *FABP1* in the livers of fasted *Ddah1*^HKO^ mice repressed the AMPK/mTOR/autophagy signaling axis, which was consistent with a previous report [27]. In contrast, inhibiting AMPK had no effect on FABP1 expression. These observations indicate that FABP1 acts as an upstream regulator of AMPK in DDAH1-mediated modulation of hepatic lipid metabolism under fasting conditions.

Our previous work demonstrated that systemic or hepatocyte-specific deletion of *Ddah1* aggravates the HFD-induced NAFLD-like phenotype in mice [13, 28]. Combined with the current findings, this suggests that the regulatory role of DDAH1 in lipid metabolism is nutrient status dependent. The key distinction between these two contexts lies in the drivers of steatosis: HFD-induced steatosis is primarily driven by lipid overload, inflammation, and oxidative stress, and DDAH1 protects the liver by suppressing these pathways [13, 28]; in contrast, fasting-induced steatosis arises from adaptive lipid mobilization and transient imbalance between lipid uptake and clearance, and DDAH1 promotes steatosis by regulating FABP1-mediated fatty acid uptake and AMPK-dependent autophagy. This nutrient-dependent duality highlights the complexity of DDAH1’s metabolic functions and underscores the importance of studying metabolic regulators in diverse physiological contexts.

In conclusion, this study identifies DDAH1 as a novel regulator of hepatic lipid metabolism under fasting conditions, which ­balances lipid uptake and degradation by coordinating FABP1 expression and autophagic signaling. These findings not only advance our understanding of the hepatic metabolic pathways governing adaptation to nutrient deprivation but also imply that targeting the DDAH1–FABP1–autophagy axis may represent a new strategy for managing metabolic liver diseases characterized by dysregulated lipid homeostasis.

### Limitations of the study

This article has two limitations. First, the mechanistic evidence for DDAH1–FABP1 regulation remains indirect. While AAV-mediated *FABP1* overexpression partially reversed the phenotype of *Ddah1*^HKO^ mice, we did not include reciprocal experiments such as *FABP1* knockdown in *DDAH1*-overexpressing mice. The molecular mechanism by which DDAH1 regulates FABP1 (e.g., via transcriptional control or protein stability) was not directly demonstrated. Second, the AMPK/mTOR/autophagy findings rely primarily on pharmacologic inhibition. The effects of DDAH1 on autophagy were largely inferred from treatment with the AMPK inhibitor Compound C. Genetic models, such as hepatocyte-specific *AMPKα* knockout mice, were not used to confirm causality.

## Materials and methods

Complete details of the reagents and primary antibodies used are listed in Supplementary Table S1 for reproducibility.

### Mice and experimental design

*Ddah1* ^HKO^ mice were generated as previously described [13], with modifications to align with fasting-induced metabolic adaptation detection. The 8–10-week-old male littermates (*Ddah1*^f/f; alb-ERT2-cre/+^ as *Ddah1*^HKO^, *Ddah1*^f/f;+/+^ as controls) were selected to minimize sex-related metabolic variability, and Cre recombinase was activated via 5 consecutive days of tamoxifen (50 mg/kg, intraperitoneally) injection. *Ddah1*^HKO^ and *Ddah1*^f/f^ mice were assigned randomly to three groups: fed group (Fed), fasted group (Fasted, 24-h fast), and refed group (Refed, 6-h refeeding after a 24-h fast).

AAV8 vectors for liver-specific overexpression of target genes were constructed by Vigene Biosciences, Inc. (Shandong, China). These vectors utilized the TBG promoter to control the expression of genes encoding a histidine tag (His), human *DDAH1* (h*DDAH1*), and human *FABP1* (h*FABP1*). To achieve hepatic overexpression of DDAH1 or FABP1 in mice, AAV8-TBG-h*DDAH1* or AAV8-TBG-h*FABP1* was administered via tail vein injection (1.1 × 10^12^ viral genome copies per mouse). Four weeks after injection, mice were selected for follow-up experiments.

### Histological assessment

Fresh liver specimens were promptly subjected to fixation in a 4% paraformaldehyde solution for 24 h, dehydrated through a graded ethanol series, cleared in xylene, and infiltrated with molten paraffin before being embedded in paraffin blocks. These blocks were subsequently sectioned into 5 μm-thick slices using a microtome for histological examination. H&E staining was used to visualize architectural changes in the liver. Frozen liver sections (4-μm thick) were stained with Oil Red O to assess lipid accumulation.

### RNA-seq analysis

Total RNA was isolated from the livers of fasted mice. Following RNA quality validation, the samples were further purified and used for cDNA library preparation. Sequencing was carried out on the BGISEQ500 platform (BGI-Shenzhen, China). DEGs were identified using the approach described previously [29]. KEGG pathway enrichment analysis was conducted using the phyper function in R, with *P*-values adjusted to control the false discovery rate and *Q*-values computed. Pathways were considered significantly enriched at *Q* ≤ 0.05.

### Lipidomics assay

A 10-mg liver tissue sample was mixed with 400 μL distilled water and steel beads, vortexed for 60 s, and then sonicated in an ice bath for 5 min. This procedure was repeated three times. Next, 10 μL of the tissue homogenate was mixed with 190 μL distilled water and 480 μL internal standard-containing extraction buffer (methyl tert-butyl ether:methanol = 5:1), vortexed, and sonicated in an ice bath for 10 min. After centrifugation at 3000 *g* for 15 min, the supernatant (approximately 250 μL) was collected, and the remaining precipitate was re-extracted twice with 250 μL methyl tert-butyl ether. The supernatants from the three extractions were combined and vacuum-dried at 37 °C. The dried metabolites were dissolved in 200 μL of a solvent mixture (dichloromethane:methanol:water = 60:30:4.5). After vortexing, sonication, and centrifugation at 13,000 *g* for 15 min at 4 °C, the supernatant was collected for subsequent ultra-high-performance liquid chromatography-tandem mass spectrometry analysis.

### Western blot analysis

For western blot analysis, liver tissues (pulverized under liquid nitrogen to preserve protein phosphorylation) or HepG2 cells (scraped in cold PBS) were homogenized in ice-cold lysis buffer (50 mmol/L Tris-HCl, pH 7.4, 150 mmol/L NaCl, 1% Triton X-100, 100 μg/mL PMSF, and 1× protease/phosphatase inhibitor cocktail) for 1 min, and then incubated on ice for 30 min to ensure complete lysis—this step was optimized to maintain AMPK/mTOR signaling protein activity, a key focus of this article.

### Statistical analysis

All experimental data are presented as “mean ± standard error of the mean (mean ± SEM)”. Statistical analysis was performed using a two-tailed Student’s *t*-test or two-way analysis of variance (two-way analysis of variance) with Tukey’s correction. A *P*-value < 0.05 was considered statistically significant. The significance ­levels are indicated as follows: *P < 0.05, ^**^P < 0.01, ^***^P < 0.001.

## Supplementary Material

loaf042_Supplementary_Data

## Data Availability

All data generated in this article are available from the corresponding author upon reasonable request.
