# Supplementary material for: Hepatocyte-specific DDAH1 regulates fasting-induced hepatic lipid metabolism via modulating FABP1 expression and AMPK/mTOR-mediated autophagy
Source: Life Metab. 2025 Dec 4;5(2):loaf042. doi: 10.1093/lifemeta/loaf042 (PMC13124279; doi:10.1093/lifemeta/loaf042)
Supplement: loaf042_Supplementary_Data [file loaf042_supplementary_data.docx]

**Supplementary Material**

**Supplementary Table S1** Information for antibodies and reagents.

| **Reagent or resource** | **Source** | **Identifier** |
| --- | --- | --- |
| **Antibodies** | | |
| ACOX1 | Proteintech Group | Cat# 83731-2-RR |
| AMPKα | Cell Signaling Technology | Cat# 2532S |
| ATGL | Cell Signaling Technology | Cat# 2138S |
| Beta Tubulin | Sino Biological Inc | Cat#100109-MM05T |
| CD36 | Sino Biological Inc | Cat# 80263-T48 |
| CIDEA | Sino Biological Inc | Cat# 100879-T32 |
| CIDEC | Signalway Antibody | Cat# 36353 |
| DDAH1 | Signalway Antibody | Cat# 37368 |
| FABP1 | Sino Biological Inc | Cat# 12353-MM06 |
| FAS | Cell Signaling Technology | Cat# 3180S |
| LC3A/B | Cell Signaling Technology | Cat# 4108S |
| mTOR | Cell Signaling Technology | Cat# 2972S |
| Phospho-AMPKα(Thr172) | Cell Signaling Technology | Cat# 2535S |
| PLIN2 | Cell Signaling Technology | Cat# 45535S |
| Phospho-mTOR(Ser2448) | Cell Signaling Technology | Cat# 2971S |
| PPAR-alpha | Proteintech Group | Cat# 66826-1-IG |
| PPAR-gamma | Cell Signaling Technology | Cat# 2435S |
| SQSTM1/p62 | Cell Signaling Technology | Cat# 5114S |
| **Chemicals and critical commercial assays** | | |
| DMEM | Gibco | Cat# 11965175 |
| FBS | Sigma-Aldrich | Cat# 102567214 |
| Oil red O | Sigma-Aldrich | Cat# O0625 |
| TRIzol reagent | Invitrogen | Cat# 15596026 |
| jetPRIME Transfection | Polyplus | Cat# 0000002940 |
| BODIPY™ FL C16 | Thermo Fisher Scientific Inc. | Cat# D3821 |
| Protease inhibitor | Roche | Cat# 04693124001 |
| phosphatase inhibitor | Roche | Cat# 4906837001 |
| Ketone Body Content Assay Kit | Solarbio Science & Technology Co., Ltd | Cat# BC5065 |
| Serum TC Kit | Nanjing Jiancheng Bioengineering Institute | Cat# A111-1-1 |
| Serum TAG kit | Nanjing Jiancheng Bioengineering Institute | Cat# A110-1-1 |
| Serum NEFA kit | Runzekang Biotechnology | Cat# Rzk-1766 |
| Serum Insulin Kit | American Laboratory Products Company | Cat# 80-INSMSU-E01 |
| Liver cholesterol Kit | Applygen Technologies Inc | Cat# E1015-105 |
| Liver TAG Kit | Applygen Technologies Inc | Cat# E1013-105 |
| SYBR® Premix Ex Taq™ II Kit | TaKaRa | Cat# RR820DS |
| PrimeScript RT Reagent Kit | TaKaRa | Cat# RR036B |
| BCA Protein Assay Kit | Beyotime Biotechnology | Cat# P0012S |


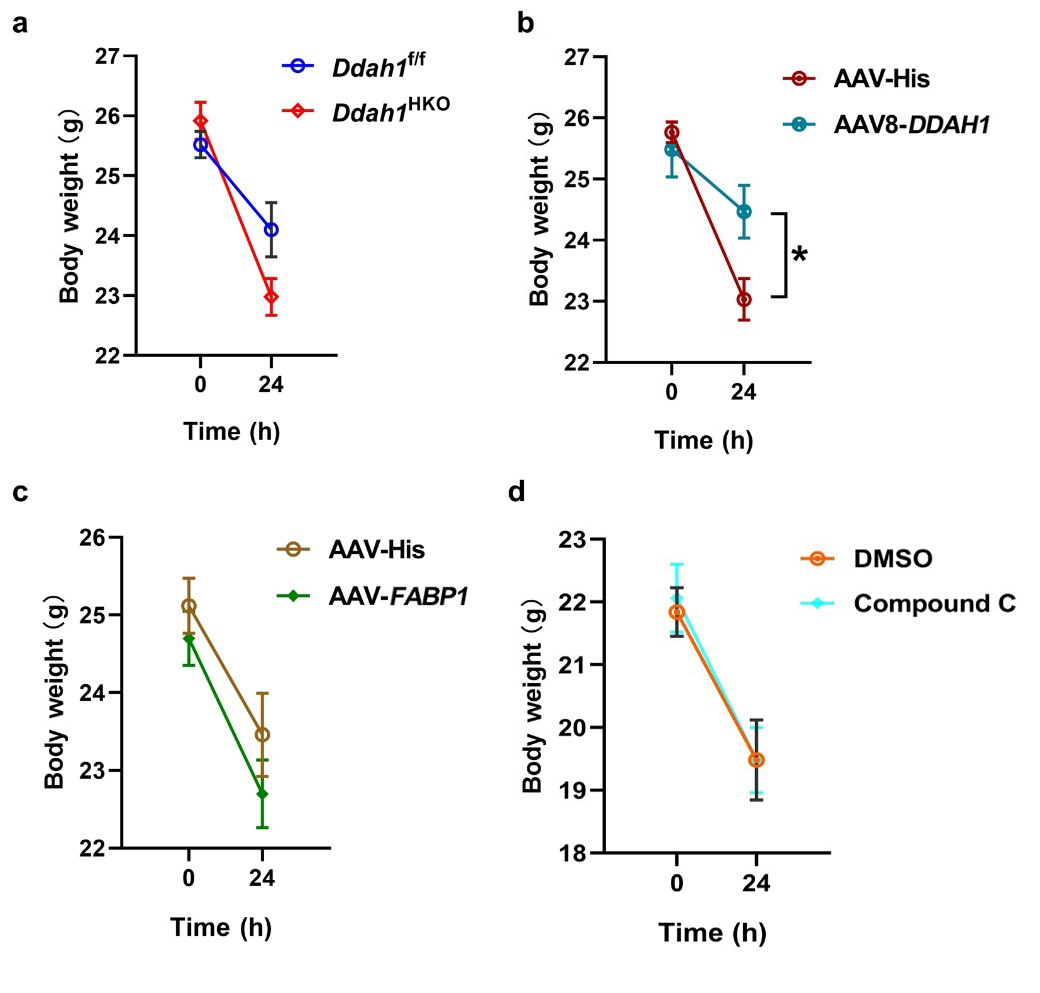


**Supplementary Figure S1** Fasting for 24 h induces bodyweight changes in different groups. (a) The changes in bodyweight recorded after *Ddah1*^f/f^ and *Ddah1*^HKO^ mice were fasted for 24 h. (b) The bodyweight of C57BL/6 mice with AAV8-His or AAV8-*DDAH1* injection before and after 24 h fasting. (c) The 24 h-fasting-induced changes in bodyweight of *Ddah1*^HKO^ mice with AAV8-His or AAV8-*FABP1* injection. (d) The changes in bodyweight of *Ddah1*^HKO^ mice treated with DMSO or 10 mg/Kg compound C and then fasted for 24 h. The values are presented as means ± SEM. ^*^*P* < 0.05.


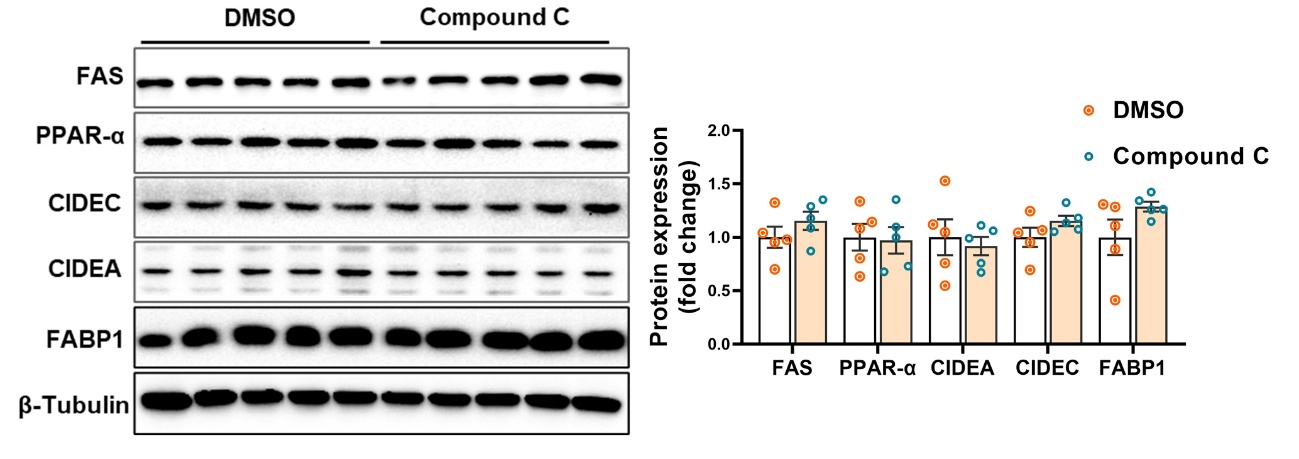


**Supplementary Figure S2** Inhibition of AMPK has no significant effect on the expression of lipid metabolism related proteins. Liver lysates were examined by western blot analysis. The values are presented as means ± SEM.
